# Supplementary material for: Characterisation of Novel Angiogenic and Potent Anti-Inflammatory Effects of Micro-Fragmented Adipose Tissue
Source: Int J Mol Sci. 2021 Mar 23;22(6):3271. doi: 10.3390/ijms22063271 (PMC8004757; doi:10.3390/ijms22063271)
Supplement: Supplementary file 1 [file ijms-22-03271-s001.pdf]

Supplementary Table 1:

Table 1: Quantikine cytokines chosen for examination by multiplex:

MIG	Eotaxin	GRO-a
MIP-1b	Basic FGF	HGF
IL-6	VEGF	IL-3
IFN-g	PDGF-BB	IL-15
IL-1Ra	IP-10	IL-7
IL-12(p40)	IL-13	IL-12(p70)
IL-5	IL-4	IL-17
GM-CSF	MCP-1	IL-9
MIF	IL-8	IL-1B
TNF-a	MIP-1a	IL-2
RANTES	IL-10	G-CSF

Supplementary Tables2 -heat maps:

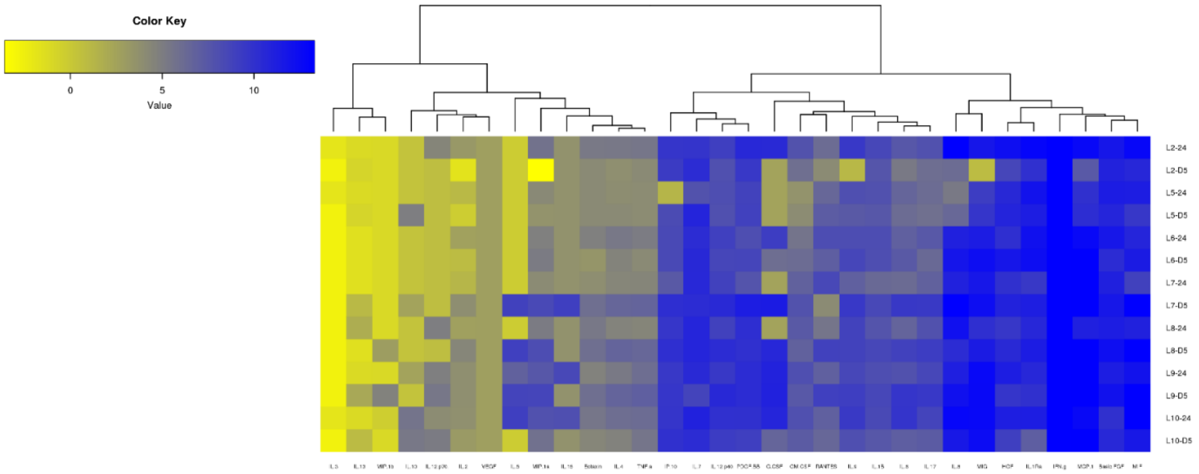

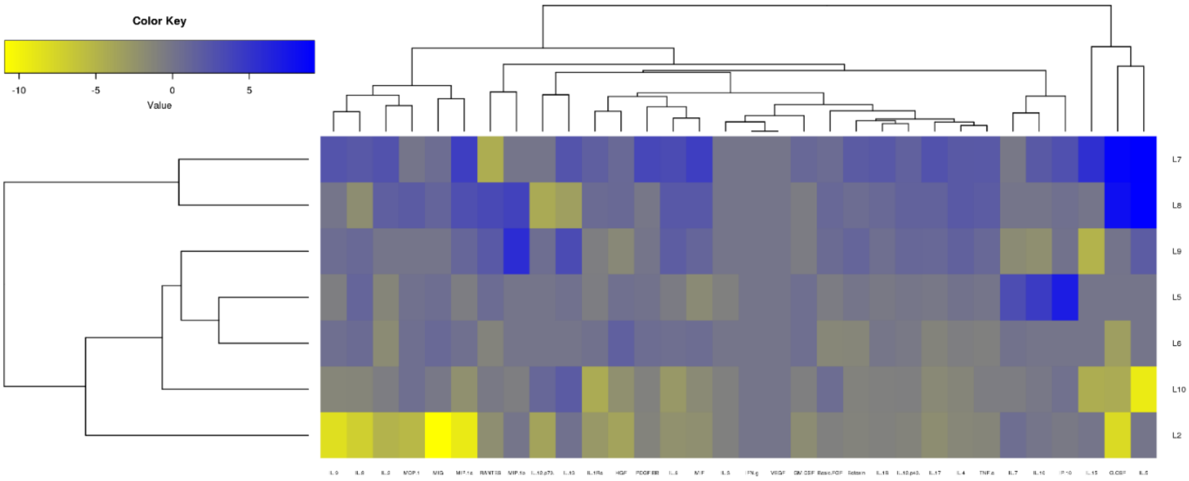

Color Key
-10
-5
0
5
Value
L7
L8
L9
L5
L6
L10
L2

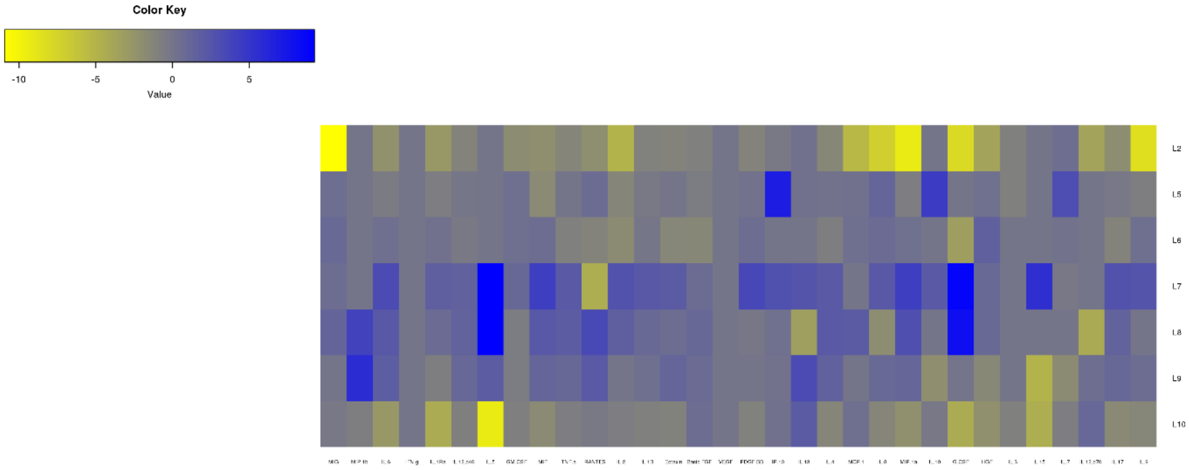

Color Key
-10
-5
0
5
Value
L2
L5
L6
L7
L8
L9
L10

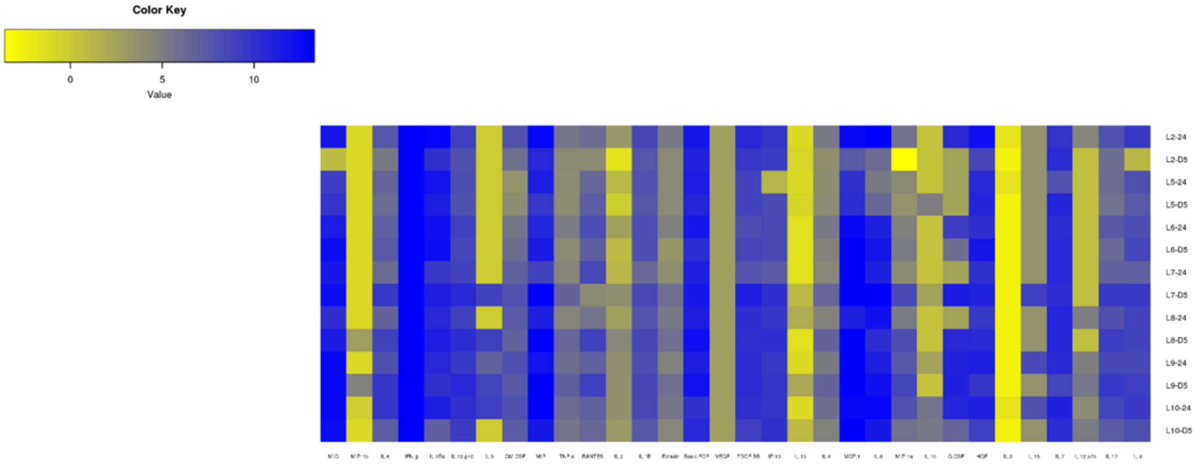

Color Key
0
5
10
Value
L2-24
L2-D5
L5-24
L5-D5
L6-24
L6-D5
L7-24
L7-D5
L8-24
L8-D5
L9-24
L9-D5
L10-24
L10-D5

Raw data: see excel file

Location Sample MIG MIP-1b IL-6 IFN-g IL-1Ra IL-12(p40) IL-5 18(1,B3) L2-24 3494.888 181.9787 6889.543 < 0.87554931640625 6404.943 < 17.4192504882813 363.4811 26(1,B4) L2-24 3669.187 181.2192 8529.502 < 0.87554931640625 7771.913 121.7885 406.358 19(1,C3) L2-D5 5227.929 710.864 2449.928 488.1342 24918.06 < 17.4192504882813 363.4811 27(1,C4) L2-D5 5477.778 711.9202 2521.389 467.3249 24233.05 < 17.4192504882813 380.8994 20(1,D3) L7-D5 6631.483 222.8003 1630.396 < 0.87554931640625 3811.046 < 17.4192504882813 253.3287 28(1,D4) L7-D5 6763.9 245.6096 1717.131 222.5566 4453.035 223.6833 298.929 21(1,E3) L9-D5 6814.559 848.6257 2755.952 479.1334 18203.55 223.6833 327.3704 22(1,F3) L9-D5 6871.833 961.4687 2522.845 625.9172 17631.59 < 17.4192504882813 336.5745 23(1,G3) L10-D5 5287.722 119.3977 121.8334 < 0.87554931640625 2948.568 < 17.4192504882813 98.32731 24(1,H3) L10-D5 5928.997 150.4671 144.8347 < 0.87554931640625 3341.276 162.9371 263.8289 29(1,E4) D5-D5 < 1.841064453125 34.17547 562.0548 < 0.87554931640625 782.3967 47.06451 133.9773 37(1,E5) D5-D5 50.13477 12.00593 574.4043 < 0.87554931640625 754.3912 452.1401 98.32731 30(1,F4) D3-D5 127.3955 102.3436 2254.683 135.2064 1591.623 314.344 225.9138 38(1,F5) D3-D5 181.0548 68.56973 1760.144 < 0.87554931640625 1235.258 482.1031 190.1211 35(1,C5) L6-D5 2800.896 477.8417 2759.956 530.4692 > 103481 482.1031 389.47 40(1,H5) L4-D5 2985.083 180.4584 447.6313 < 0.87554931640625 6337.959 < 17.4192504882813 < 3.6416015625 32(1,H4) L4-D5 3437.913 155.3605 643.5812 < 0.87554931640625 10779.93 < 17.4192504882813 133.9773 36(1,D5) L8-D5 6596.352 856.8833 1031.239 445.1728 24062.58 538.6948 380.8994 31(1,G4) L5-D5 1126.223 100.3612 1617.336 < 0.87554931640625 2068.133 47.06451 208.4883 64(1,H8) L3-D5 1573.084 < 0.52490234375 437.6861 < 0.87554931640625 778.9095 < 17.4192504882813 < 3.6416015625 65(1,A9) D9-24 853.0882 63.52005 2707.071 < 0.87554931640625 1550.435 < 17.4192504882813 98.32731 66(1,B9) D10-24 662.0411 < 0.52490234375475.8 < 0.87554931640625 125.5983 < 17.4192504882813 < 3.6416015625 67(1,C9) L3-24 < 1.841064453125 < 0.52490234375 < 0.36083984375 < 0.87554931640625 < 6.31597900390625 < 17.4192504882813 < 3.6416015625 68(1,D9) L4-24 < 1.841064453125 < 0.52490234375 13.89529 < 0.87554931640625 316.3233 < 17.4192504882813 < 3.6416015625 69(1,E9) L5-24 < 1.841064453125 37.98758 1102.424 < 0.87554931640625 1655.83 < 17.4192504882813 202.4814 73(1,A10) L6-24 527.9942 98.35816 3698.933 < 0.87554931640625 2413.622 < 17.4192504882813 247.9862 89(1,A12) L6-24 682.6704 96.33352 4565.228 < 0.87554931640625 2747.439 162.9371 237.0989 74(1,B10) L7-24 853.0882 74.5832 2237.881 < 0.87554931640625 771.9236 < 17.4192504882813 214.3913 90(1,B12) L7-24 861.8977 67.3291 2965.815 < 0.87554931640625 820.5123 272.1847 149.3507 75(1,C10) L8-24 2853.728 133.6148 4520.036 < 0.87554931640625 1968.478 352.4574 294.0535 91(1,C12) L8-24 2672.216 107.2158 4681.116 < 0.87554931640625 1719.437 223.6833 279.1619 76(1,D10) L9-24 5173.184 168.103 5412.863 < 0.87554931640625 2885.291 223.6833 258.6085 92(1,D12) L9-24 5590.828 160.1888 5443.283 < 0.87554931640625 2834.425 47.06451 289.1351 77(1,E10) L10-24 3540.739 79.19601 744.4313 < 0.87554931640625 511.9402 314.344 75.87806 93(1,E12) L10-24 3259.472 67.3291 727.9754 < 0.87554931640625 468.9897 420.7785 75.87806 94(1,F12) D6 798.931 85.84238 5979.85 < 0.87554931640625 472.9328 223.6833 318.0348 86(1,F11) D6 922.0113 97.34861 5669.37 < 0.87554931640625 527.3399 47.06451 359.0649 95(1,G12) D7 722.7651 64.80499 3941.81 < 0.87554931640625 1321.867 272.1847 202.4814 87(1,G11) D7 789.6731 72.21356 3796.281 < 0.87554931640625 1574.003 < 17.4192504882813 268.9928 96(1,H12) D8 662.0411 < 0.52490234375 902.2609 < 0.87554931640625 624.9776 162.9371 < 3.6416015625 88(1,H11) D8 826.2974 19.71684 1191.003 < 0.87554931640625 817.0653 272.1847 75.87806 IL-1Ra 8000

GM-CSF MIF TNF-a RANTES IL-2 IL-1B Eotaxin Basic FGF VEGF < 0.4989013671875 969881 449.2696
200.5286 35.49832 6.999928 38.54729 3182.935 < 6.7840576171875 < 0.4989013671875 38676670
676.266 237.3444 52.39376 11.60903 38.54729 4053.425 < 6.7840576171875 < 0.4989013671875
2862639 1394.55 221.2926 93.87496 12.47615 56.06543 3288.07 < 6.7840576171875 <
0.4989013671875 1949872 1517.719 229.3759 110.2621 17.4299 65.98382 3544.275 <
6.7840576171875 < 0.4989013671875 357953.5 637.6245 233.3741 48.19125 18.22216 25.39235
2450.131 < 6.7840576171875 < 0.4989013671875 1243431 797.0381 294.0458 81.5173 10.2793
25.39235 2660.234 < 6.7840576171875 < 0.4989013671875 953808.9 1172.514 268.1984 60.76336
15.41125 42.86703 1110.169 < 6.7840576171875 1.105628 375646.3 1034.354 249.0968 79.45137
11.60903 46.89097 953.6432 < 6.7840576171875 < 0.4989013671875 84736.82 569.9021 121.509
35.49832 6.000453 11.94631 1364.764 < 6.7840576171875 < 0.4989013671875 169859.4 701.3332
183.2635 56.58414 11.60903 31.23212 1844.486 < 6.7840576171875 7.496607 11157.98 299.5824
32.43453 26.94682 2.067605 25.39235 2980.527 < 6.7840576171875 34.53814 11329.33 341.5662
48.74981 39.74506 6.000453 33.81469 3593.285 < 6.7840576171875 24.2665 62492.75 415.6221
11.23465 18.29452 6.504614 31.23212 2083.299 < 6.7840576171875 17.60363 41665.78 115.3002
63.05056 22.63566 1.389068 17.76942 1982.164 < 6.7840576171875 7.496607 5382448 1285.504
110.9121 89.76266 21.31716 61.14116 5211.262 < 6.7840576171875 < 0.4989013671875 14551.96
341.5662 99.88317 9.485403 1.389068 < 0.11920166015625 1336.905 < 6.7840576171875 <
0.4989013671875 500192.3 597.5476 131.7405 13.91554 < 0.31085205078125 25.39235 1002.116
< 6.7840576171875 26.06204 8642978 1517.719 178.8438 128.5935 14.17065 61.14116 4322.157 <
6.7840576171875 < 0.4989013671875 673500.6 481.2691 160.6913 22.63566 6.504614 40.74997
2636.235 < 6.7840576171875 < 0.4989013671875 5950.76 < 3.2933349609375 99.88317 <
1.55828857421875 69.07613 < 0.11920166015625 1465.315 < 6.7840576171875 <
0.4989013671875 18199.69 < 3.2933349609375 < 0.966064453125 0.270646 < 0.31085205078125 <
0.11920166015625 956.7319 < 6.7840576171875 < 0.4989013671875 7604.875 < 3.2933349609375
< 0.966064453125 < 1.55828857421875 < 0.31085205078125 < 0.11920166015625 414.9551 <
6.7840576171875 < 0.4989013671875 < 3.05877685546875 < 3.2933349609375 < 0.966064453125
< 1.55828857421875 < 0.31085205078125 < 0.11920166015625 < 3.9283447265625 <
6.7840576171875 < 0.4989013671875 472.8179 < 3.2933349609375 < 0.966064453125 <
1.55828857421875 < 0.31085205078125 < 0.11920166015625 456.2429 < 6.7840576171875 <
0.4989013671875 284369.1 252.2065 63.05056 18.29452 < 0.31085205078125 17.76942 1975.383
< 6.7840576171875 < 0.4989013671875 300088.9 252.2065 < 0.966064453125 16.11041 <
0.31085205078125 17.76942 2366.506 < 6.7840576171875 < 0.4989013671875 890338.4 299.5824
22.89695 16.11041 3.86798 17.76942 3061.866 < 6.7840576171875 < 0.4989013671875 220012
252.2065 < 0.966064453125 18.29452 1.389068 17.76942 1378.519 < 6.7840576171875 <
0.4989013671875 274507.9 252.2065 32.43453 13.91554 < 0.31085205078125 17.76942 2203.909
< 6.7840576171875 < 0.4989013671875 1816816 624.4369 88.33095 35.49832 12.04438 40.74997
4186.824 < 6.7840576171875 < 0.4989013671875 765151.2 415.6221 11.23465 31.23315 <
0.31085205078125 15.2047 2799.378 < 6.7840576171875 < 0.4989013671875 377276.3 415.6221
63.05056 18.29452 3.294748 21.92888 1099.209 < 6.7840576171875 < 0.4989013671875 392863.7
398.0604 76.12266 18.29452 < 0.31085205078125 < 0.11920166015625 1480.448 <
6.7840576171875 < 0.4989013671875 202139.3 496.7438 110.9121 31.23315 4.422214 17.76942
2313.355 < 6.7840576171875 < 0.4989013671875 191515.2 481.2691 110.9121 13.91554 <
0.31085205078125 11.94631 1960.057 < 6.7840576171875 < 0.4989013671875 67255.3 195.5099
32.43453 18.29452 1.389068 28.44923 1250.293 < 6.7840576171875 < 0.4989013671875 81005.49
160.5006 63.05056 13.91554 1.389068 < 0.11920166015625 1077.015 < 6.7840576171875 <
0.4989013671875 54785.36 379.922 < 0.966064453125 11.70807 2.067605 19.96691 2055.526 <
6.7840576171875 < 0.4989013671875 39746.13 195.5099 < 0.966064453125 0.270646 <

0.31085205078125 6.289433 2185.231 < 6.7840576171875 < 0.4989013671875 47688.11 < 3.2933349609375 < 0.966064453125 4.976093 < 0.31085205078125 11.94631 1660.894 < 6.7840576171875 < 0.4989013671875 76489.47 < 3.2933349609375 11.23465 9.485403 1.389068 17.76942 1855.237 < 6.784057617

PDGF-BB IP-10 IL-13 IL-4 MCP-1 IL-8 MIP-1a IL-10 G-CSF 1633.909 655.873 < 0.34893798828125 37.57635 4001.901 8775.887 50.10385 < 1.339843751478.87 1544.907 1063.878 0.589362 42.38943 > 9267 10322.27 51.28482 < 1.339843751370.439 2653.153 1125.38 2.578044 89.91579 > 9267 9131.147 349.8741 < 1.339843752556.071 2474.899 1238.264 1.582976 96.78729 > 9267 10359.75 352.6949 10.01168 3081.448 747.136 655.873 < 0.34893798828125 32.50378 > 9267 2288.503 185.8849 < 1.339843752019.634 1858.286 848.1499 0.589362 42.38943 > 9267 2491.222 182.8606 10.01168 1880.777 543.3672 655.873 0.589362 59.86721 > 9267 6988.46 240.4878 < 1.339843751648.255 1574.908 1063.878 < 0.34893798828125 67.85865 3900.737 7857.468 240.8049 95.30238 2041.848 17.92801 1125.38 < 0.34893798828125 14.57604 > 9267 2857.153 50.89429 < 1.33984375< 5.82110595703125 1078.371 998.0212 3.573964 37.57635 > 9267 3339.897 61.02829 79.57558 185.0227 699.6082 < 2.5184326171875 < 0.34893798828125 21.20965 43.00182 32.53956 < 0.084228515625 10.01168 < 5.82110595703125 < 3.24786376953125 316.6136 1.085914 46.99398 76.8148 70.52524 6.499746 < 1.33984375< 5.82110595703125 880.0608 316.6136 2.08038 46.99398 1046.194 206.0594 20.86728 52.51559 < 5.82110595703125 274.763 < 2.5184326171875 5.068938 32.50378 878.8211 157.1661 19.86123 < 1.33984375< 5.82110595703125 1078.371 1125.38 < 0.34893798828125 103.47 > 9267 1283.409 288.4746 < 1.339843751639.798 1001.889 316.6136 < 0.34893798828125 14.57604 2654.643 4147.916 174.7894 < 1.339843751780.427 1187.379 < 2.5184326171875 1.582976 6.318841 > 9267 5119.417 153.329 < 1.33984375445.4403 1290.693 848.1499 4.570491 103.47 > 9267 4550.687 415.0871 < 1.339843752091.858 1222.396 848.1499 3.573964 24.22263 2218.544 4735.624 35.59889 < 1.33984375< 5.82110595703125 274.763 < 2.5184326171875 < 0.34893798828125 < 0.220947265625 1895.868 2596.531 57.16192 < 1.33984375< 5.82110595703125 < 3.24786376953125 < 2.5184326171875 < 0.34893798828125 < 0.220947265625 3778.333 4759.304 30.05923 < 1.33984375< 5.82110595703125 < 3.24786376953125 < 2.5184326171875 < 0.34893798828125 < 0.220947265625 1152.987 1524.171 < 0.084228515625 < 1.33984375< 5.82110595703125 < 3.24786376953125 < 2.5184326171875 < 0.34893798828125 < 0.220947265625 < 0.56561279296875 5.147092 < 0.084228515625 < 1.33984375< 5.82110595703125 < 3.24786376953125 < 2.5184326171875 < 0.34893798828125 < 0.220947265625 < 0.56561279296875 < 0.92919921875 < 0.084228515625 < 1.33984375525.049 747.136 655.873 0.589362 14.57604 166.8161 70.52524 < 0.084228515625 < 1.33984375< 5.82110595703125 650.062 < 2.5184326171875 < 0.34893798828125 10.77395 932.2247 20.63866 16.46642 < 1.33984375< 5.82110595703125 274.763 < 2.5184326171875 0.589362 21.20965 1133.744 52.75465 21.35204 < 1.33984375< 5.82110595703125 1001.889 316.6136 0.589362 14.57604 1196.919 116.7257 11.23183 62.19078 < 5.82110595703125 < 3.24786376953125 316.6136 0.589362 21.20965 1309.536 52.75465 12.95176 < 1.33984375< 5.82110595703125 274.763 655.873 < 0.34893798828125 61.90305 > 9267 2298.657 29.3773 < 1.33984375575.8405 274.763 < 2.5184326171875 < 0.34893798828125 18.0152 4168.225 2002.707 26.85034 < 1.33984375644.6407 422.2065 655.873 < 0.34893798828125 21.20965 > 9267 3670.875 32.64094 < 1.33984375< 5.82110595703125 422.2065 < 2.5184326171875 < 0.34893798828125 27.09312 > 9267 3788.858 39.35869 < 1.33984375120.6319 < 3.24786376953125 < 2.5184326171875 < 0.34893798828125 42.38943 > 9267 2071.473 19.338 < 1.33984375< 5.82110595703125 422.2065 316.6136 < 0.34893798828125 < 0.220947265625 > 9267 1984.054 15.16142 < 1.33984375<

5.82110595703125 < 3.24786376953125 < 2.5184326171875 0.093868 6.318841 1239.547 116.7257 15.82776 < 1.33984375< 5.82110595703125 179.5121 316.6136 0.093868 21.20965 1227.792 182.183 14.46349 < 1.33984375< 5.82110595703125 < 3.24786376953125 < 2.5184326171875 < 0.34893798828125 14.57604 2234.706 1286.511 15.82776 < 1.33984375< 5.82110595703125 < 3.24786376953125 < 2.5184326171875 < 0.34893798828125 14.57604 2289.379 1585.161 17.08055 < 1.33984375< 5.82110595703125 < 3.24786376953125 < 2.5184326171875 < 0.34893798828125 < 0.220947265625 1847.817 1462.325 < 0.084228515625 < 1.33984375< 5.82110595703125 < 3.24786376953125 316.6136 < 0.34893798828125 6.318841 2160.583 1870.82 7.959499 < 1.33984375< 5.821105957031

GRO-a HGF IL-3 IL-15 IL-7 IL-12(p70) IL-17 IL-9 N/A 4500.806 < 0.15447998046875 < 12.308837890625 < 3.07269287109375 < 1.68359375231.2148 541.2856 N/A 5732.685 0.401578 < 12.308837890625 1806.832 43.48581 257.6046 945.7721 N/A 1973.808 < 0.15447998046875 540.7268 1320.493 < 1.68359375659.2853 797.8563 N/A 1973.808 < 0.15447998046875 584.3227 1320.493 < 1.68359375742.0517 686.4498 2780 N/A 2281.675 < 0.15447998046875 242.2522 1320.493 < 1.68359375324.3336 287.7122 2386 N/A 2874.816 < 0.15447998046875 475.4774 1581.075 58.64714 439.4691 480.8756 2663 N/A 2544.488 < 0.15447998046875 < 12.308837890625 1806.832 < 1.68359375439.4691 657.996 2357 N/A 2432.537 0.401578 525.298 2372.134 31.26054 454.0869 714.6491 2539 N/A 400.2983 < 0.15447998046875 < 12.308837890625 2197.218 < 1.6835937590.14372 180.1457 2666 N/A 701.665 < 0.15447998046875 < 12.308837890625 440.8374 69.76329 213.0992 354.5641 2780 N/A 290.3695 < 0.15447998046875 < 12.308837890625 1320.493 < 1.68359375184.9905 180.1457 N/A 171.2895 0.401578 < 12.308837890625 440.8374 < 1.68359375175.3313 99.89179 N/A 1067.602 3.59326 < 12.308837890625 2694.616 58.64714 175.3313 370.8355 N/A 1023.381 2.285841 < 12.308837890625 1320.493 < 1.68359375< 2.79693603515625 253.0173 2511 N/A 1409.981 < 0.15447998046875 < 12.308837890625 1806.832 < 1.68359375606.7428 450.0519 2578 N/A 604.6803 < 0.15447998046875 < 12.308837890625 440.8374 < 1.68359375213.0992 < 2.22259521484375 2489 N/A 504.5329 < 0.15447998046875 < 12.308837890625 < 3.07269287109375 43.48581 213.0992 386.9529 2212 N/A 888.3554 < 0.15447998046875 < 12.308837890625 440.8374 43.48581 773.1274 585.6387 2332 N/A 796.0845 < 0.15447998046875 < 12.308837890625 1806.832 31.26054 231.2148 450.0519 2307 N/A 105.4963 < 0.15447998046875 < 12.308837890625 < 3.07269287109375 < 1.68359375< 2.79693603515625 < 2.22259521484375 2285 N/A 604.6803 < 0.15447998046875 < 12.308837890625 < 3.07269287109375 < 1.68359375< 2.79693603515625 < 2.22259521484375 2584 N/A 171.2895 < 0.15447998046875 < 12.308837890625 < 3.07269287109375 < 1.68359375< 2.79693603515625 < 2.22259521484375 2418 N/A < 9.64349365234375 < 0.15447998046875 < 12.308837890625 440.8374 < 1.68359375< 2.79693603515625 < 2.22259521484375 2396 N/A < 9.64349365234375 < 0.15447998046875 < 12.308837890625 < 3.07269287109375 < 1.68359375< 2.79693603515625 < 2.22259521484375 2242 N/A 400.2983 < 0.15447998046875 < 12.308837890625 1320.493 < 1.6835937565.10467 < 2.22259521484375 2566 N/A 1492.922 < 0.15447998046875 < 12.308837890625 440.8374 < 1.6835937551.43908 354.5641 2373 N/A 1492.922 0.401578 < 12.308837890625 < 3.07269287109375 < 1.6835937590.14372 180.1457 2904 N/A 1656.19 < 0.15447998046875 < 12.308837890625 2845.233 < 1.6835937565.10467 321.5172 2952 N/A 2128.832 < 0.15447998046875 < 12.308837890625 994.7167 < 1.6835937551.43908 < 2.22259521484375 2637 N/A 1326.073 < 0.15447998046875 < 12.308837890625 1320.493 < 1.68359375257.6046 321.5172 2350 N/A 978.7834 < 0.15447998046875 < 12.308837890625 1320.493 < 1.6835937590.14372 253.0173 2540 N/A 3584.243 < 0.15447998046875 < 12.308837890625 2537.345 < 1.6835937590.14372 418.7613 2993 N/A 3653.6 < 0.15447998046875

< 12.308837890625 440.8374 < 1.6835937565.10467 386.9529 2380 N/A 1154.996 < 0.15447998046875 < 12.308837890625 1320.493 < 1.68359375134.8521 99.89179 2696 N/A 888.3554 < 0.15447998046875 < 12.308837890625 1806.832 < 1.6835937590.14372 141.281 2269 N/A 1973.808 < 0.15447998046875 < 12.308837890625 < 3.07269287109375 < 1.68359375113.1818 253.0173 2262 N/A 1816.36 2.285841 < 12.308837890625 2197.218 < 1.6835937536.53322 418.7613 N/A 1409.981 < 0.15447998046875 < 12.308837890625 < 3.07269287109375 < 1.68359375< 2.79693603515625 54.18527 2355 N/A 1326.073 < 0.15447998046875 < 12.308837890625 < 3.07269287109375 < 1.68359375< 2.79693603515625 217.2504 N/A 29.14401 < 0.15447998046875 < 12.308837890625 < 3.07269287109375 < 1.68359375< 2.79693603515625 < 2.22259521484375 2582 N/A 749.1662 < 0.15447998046875 < 12.308837890625 1581.075 58.64714 < 2.79693603515625 54.18527
